# Validation of the Blended Learning Usability Evaluation–Questionnaire (BLUE-Q) through an innovative Bayesian questionnaire validation approach

**DOI:** 10.3352/jeehp.2024.21.31

**Published:** 2024-11-07

**Authors:** Anish Kumar Arora, Charo Rodriguez, Tamara Carver, Hao Zhang, Tibor Schuster

**Affiliations:** 1 Family Medicine Education Research Group, Department of Family Medicine, Faculty of Medicine & Health Sciences, McGill University, Montréal, QC, Canada; 2 Office of Education Scholarship, Department of Family and Community Medicine, University of Toronto, Toronto, ON, Canada; 3 Institute of Health Sciences Education, Faculty of Medicine & Health Sciences, McGill University, Montréal, QC, Canada; Hallym University, Korea

**Keywords:** Bayesian method, Questionnaire design, Validation study, Program evaluation, Medical education

## Abstract

**Purpose:**

The primary aim of this study is to validate the Blended Learning Usability Evaluation–Questionnaire (BLUE-Q) for use in the field of health professions education through a Bayesian approach. As Bayesian questionnaire validation remains elusive, a secondary aim of this article is to serve as a simplified tutorial for engaging in such validation practices in health professions education.

**Methods:**

A total of 10 health education-based experts in blended learning were recruited to participate in a 30-minute interviewer-administered survey. On a 5-point Likert scale, experts rated how well they perceived each item of the BLUE-Q to reflect its underlying usability domain (i.e., effectiveness, efficiency, satisfaction, accessibility, organization, and learner experience). Ratings were descriptively analyzed and converted into beta prior distributions. Participants were also given the option to provide qualitative comments for each item.

**Results:**

After reviewing the computed expert prior distributions, 31 quantitative items were identified as having a probability of “low endorsement” and were thus removed from the questionnaire. Additionally, qualitative comments were used to revise the phrasing and order of items to ensure clarity and logical flow. The BLUE-Q’s final version comprises 23 Likert-scale items and 6 open-ended items.

**Conclusion:**

Questionnaire validation can generally be a complex, time-consuming, and costly process, inhibiting many from engaging in proper validation practices. In this study, we demonstrate that a Bayesian questionnaire validation approach can be a simple, resource-efficient, yet rigorous solution to validating a tool for content and item-domain correlation through the elicitation of domain expert endorsement ratings.

## Graphical abstract

[Fig f2-jeehp-21-31]

## Introduction

### Background/rationale

Evaluation of educational interventions (e.g., modules, workshops, classes, courses, clerkship rotations, etc.) constitutes a major subfield within health professions education, whose scholars aim to understand what, how, and why specific outcomes emerged for learners (e.g., undergraduate students, graduate students, residents, clinicians taking part in continuing professional development programs, educators taking part in faculty development programs, etc.) [1,2]. In health professions education, the evaluative focus is often oriented on the development and implementation of interventions (i.e., process evaluation) and the effectiveness of interventions (i.e., outcome evaluations) [3]. Numerous frameworks and models have been developed to guide evaluation in this context [2,3]. However, evaluations for blended learning programs remain haphazard [4]. Blended learning programs are educational interventions that combine synchronous (e.g., face-to-face real-time workshops) and asynchronous (e.g., recorded online modules) learning modalities [5]. Blended learning programs have gained significant traction over the last decade given their various benefits for learners (e.g., better control of content, sequence, pace, and time of learning) and educators (e.g., ability to split educational material with knowledge-building content in asynchronous modules and skill-building content in synchronous sessions) [5]. Notably, through a scoping review that retained 80 studies from across 25 countries, the disparity around blended learning program evaluation is made evident [4]. Evaluation terminology is often undefined and poorly conceptualized across studies [4]. Moreover, while most scholars employ questionnaires to conduct their blended learning program evaluations, less than half of these are validated, and none have been designed for the purpose of evaluating blended learning programs [4].

To address this gap, the Blended Learning Usability Evaluation–Questionnaire (BLUE-Q) was designed to comprehensively evaluate the content, the asynchronous learning modality, and the synchronous learning modality of blended learning programs across the field of health professions education [5] (Supplement 1). This tool highlights the effectiveness, efficiency, satisfaction, accessibility, organization, and learner experience of engaging with a blended learning program [4,5]. This tool must undergo a rigorous validation process to ensure that all items within the BLUE-Q are relevant, well-designed, measure what they seek to measure, and are easy to understand and interpret by end users [6]. This process is also important for ensuring that all items are associated with appropriate domains (e.g., effectiveness) and that the tool consists of only the most relevant and necessary items. However, this multi-phase process can be complex, costly, and time-consuming [6]. Some have proposed adopting a Bayesian validation approach to assist scholars and educators in engaging in rigorous validation practices [7]. While this approach has been theorized as more efficient and cost-effective at measuring the content validity of questionnaires [8], its application in health professions education has been elusive [9].

### Objectives

Thus, this study aims to present the validation of the BLUE-Q through the Bayesian questionnaire validation approach. Its secondary aim is to serve as a simplified tutorial of the Bayesian validation approach for health professions education-based evaluative scholars.

## Methods

### Ethics statement

Ethics approval to conduct this study was received from the McGill University Faculty of Medicine and Health Science’s Institutional Review Board (study no., A06-E42-18A). All participants signed consent forms prior to engaging in the study.

### Study design

This is a methodological study. The Bayesian questionnaire validation approach was used to initially validate the BLUE-Q. In essence, through the Bayesian questionnaire validation approach, experts are asked to provide a rating that expresses their level of endorsement (i.e., if they perceive that the respective item is appropriate for measuring the specific “latent trait” or, in other words: “construct,” “factor,” or “domain” it was designed to measure), for each item of the questionnaire. Consistently low ratings indicate that an item must be revised or removed from the tool. For further explanation of the Bayesian approach, please see the Supplement 2.

### Setting

This study was carried out in the Faculty of Medicine and Health Sciences at McGill University during the pandemic years, with data collection taking place between January 6, 2022, and March 10, 2022.

### Participants

Twelve faculty members were contacted, and 10 agreed to participate in an interviewer-administered survey to facilitate the Bayesian questionnaire validation of the BLUE-Q. To be eligible, participants must have expertise in teaching clinical, undergraduate, and graduate health professional learners using blended learning approaches.

### Variables

Participants rated how well they perceived each item in the BLUE-Q to reflect its underlying usability domain (i.e., effectiveness, efficiency, satisfaction, accessibility, organization, and user experience). This data was used to measure content validity as perceived by experts.

### Data sources/measurement

After an initial recruitment email was sent, the interviewer (A.K.A.) and participants met one-on-one via a videoconferencing call for approximately 30 minutes to complete an interviewer-administered survey. Facilitating this rating task, we asked participants: “how likely do you think the following item reflects the current usability domain it is ascribed to (e.g., effectiveness, efficiency, satisfaction, etc.)?” Participants were asked to provide their response on a 5-point scale: unlikely, possibly, moderately, likely, and very likely. These 5-points correspond to 0, 0.25, 0.5, 0.75, and 1, respectively, where 0 indicated “no item-domain correlation,” 0.25 indicated “some item-domain correlation,” 0.5 indicated “moderate item-domain correlation,” 0.75 indicated “great item-domain correlation,” and 1 indicated “perfect item-domain correlation.” A column for “I can’t judge” and another for “comments” was also provided to participants. Once all experts had completed their ratings for each item, all scores were compiled in one Excel document by item. A mean and standard deviation can then be calculated to observe overall endorsement and variability by item and respondent. A column called Alpha and another column called Beta must be made to calculate the beta prior distribution. The Alpha column refers to the sum of all rater scores per item. The Beta column refers to the number of raters minus the Alpha. The file can then be imported into R Statistical Software ver. 4.4.1 (https://www.r-project.org/). The following code can be applied:

Priors<-read.csv(“/Users/Downloads/Beta-distributions-BLUE-Q.csv”) #Note please adjust the directory according to where you saved the file

x<-seq(0,1,by=0.1)

par(mfrow=c(3,6),mar=c(3,3,3,1))

for(i in 1:54)

{

  assign(paste(“y”,i,sep=“”),dbeta(x,Priors$Alpha[i],Priors$Beta[i]))

    plot(x,get(paste(“y”,i,sep=“”)),main=paste(“Item”,i),type=“l”)

    points(x,get(paste(“y”,i,sep=“”)),pch=20,cex=2)

}

The resulting figures represent the beta prior distribution, which helps visualize the central tendency (i.e., distribution mode) and consistency (i.e., distribution variance) of expert ratings in a probabilistic manner. Items with left-skewed distribution suggest stronger item-domain correlations than right-skewed prior distributions. Participants were also given the option to provide qualitative comments for each item.

### Bias

For the BLUE-Q, expert input was obtained through an interviewer-administered survey that employed a visual analog rating scale. This approach to collecting expert input is beneficial as it ensures that participants understand the task well. However, a potential risk is that it may lead to social desirability bias (i.e., respondents hide their true opinions and instead provide the researcher with data they perceive as being what the researcher is looking for). To mitigate this bias, the interviewer hid their video and muted their audio after explaining the research task to the participants. Once the participants indicated they were done, the interviewer would open their video and audio to respond. Additionally, when recruiting experts for prior elicitation, a purposeful sampling approach is essential in ensuring a broad array of experiences and perspectives are considered and that undesirable systematic error (i.e., rater bias) is avoided or minimized. Thus, a broad range of experts were recruited to participate in this study.

### Study size

The beauty of the Bayesian approach is that it allows researchers to establish Prior statistics through relatively few expert participants. Bayesian Posterior statistics, however, often require far more participants. As this study only leverages expert knowledge to inform the content validity of the BLUE-Q, a sample of 10 diverse faculty members with relevant expertise was deemed sufficient. To identify experts, we reviewed all faculty members (i.e., assistant professors, associate professors, and full professors) in the Faculty of Medicine and Health Sciences. We searched for faculty who taught clinical, undergraduate, and graduate courses in the faculty using a blended learning approach for more than 2 years. Participants must have also applied some form of end-of-course evaluation to their learners or have published at least one paper on program evaluation to be included in this study. By purposefully recruiting participants with diverse teaching experiences (e.g., blended learning programs delivered to MSc and PhD students, MD and nursing students, continuing professional development programs, etc.), the generalizability of the BLUE-Q to different contexts was ascertained.

### Statistical methods

All data were prepared on Microsoft Excel version 16.89.1 (Microsoft Corp.). The beta prior distributions were conducted on R Statistical Software version 2023.06.0+421. No additional R package needs to be downloaded to conduct this analysis. After that, the first and last authors met to review quantitative results. Items with a peak distribution of 0.6 or less were removed, and those with peak distributions of 0.8 or above were kept. Meetings with the second and last author were then conducted to further choose which items would be important to keep among those with peak distribution between 0.6 and 0.8 and refine the items’ phrasing and order. Note, it was impossible to measure reliability (e.g., Cronbach’s α) and other validity measures (e.g., concurrent validity) in this study as the only data collected was experts’ endorsement ratings for each item and their qualitative comments regarding potential changes needed. Future studies about the BLUE-Q will collect and report reliability and other validity evidence, particularly through application with health professional learners.

## Results

### Participants

The 10 members consisted of 5 males and 5 females; 3 assistant professors, 2 associate professors, and 5 full professors from across the Faculty of Medicine and Health Sciences. All participants had experience teaching blended learning programs to different groups of learners (e.g., undergraduate health professional students, medical students, nursing students, graduate students, clinicians in continuing professional development programs, and health faculty in faculty development training programs). All participants also had experience completing program evaluations or publishing program evaluation-focused studies.

### Main results

Table 1 displays the expert endorsement ratings for the first 8 items of the BLUE-Q, which all intend to measure the “effectiveness” of the course content and materials for blended learning programs.

**Fig. 1.**
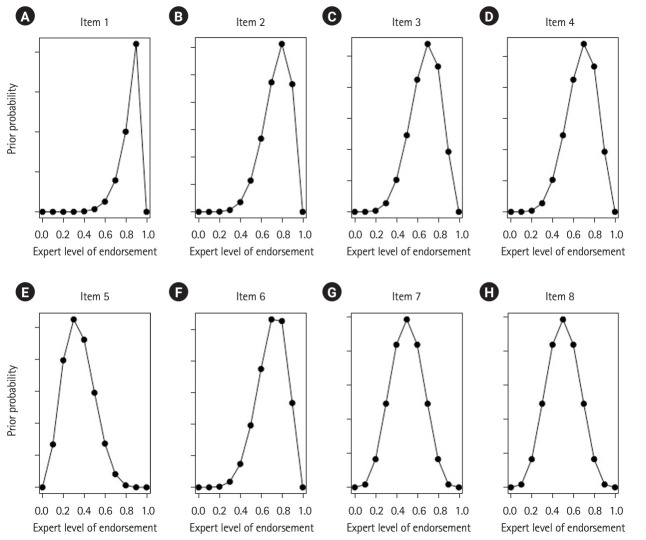
(A–H) Beta prior distributions summarizing the expert endorsements of the first 8 items of the Blended Learning Usability Evaluation–Questionnaire (BLUE-Q) measuring “effectiveness” of course content and material. The parameters of the respective beta distributions were elicited using input from 10 domain experts, summarized in Table 1.

As the variation of the numerical ratings per item indicate, there was considerable heterogeneity in the appropriateness assessment across the experts. Fig. 1 displays the resulting beta prior distributions for the same 8 items.

This figure consolidates the data distribution and clearly illustrates which items have obtained higher overall expert endorsement. Item 1 received the highest and most consistent endorsement, followed by items 2, 3, 4, and 6. Items 5, 7, and 8 showed peaks (i.e., modes) of prior distributions less or equal to 0.5, indicating experts’ hesitancy in endorsing these items. The expert rating tables (Dataset 1) and corresponding prior distribution plots for all initial BLUE-Q items by all domains (Supplements 3) are provided.

The initial version of the BLUE-Q had 54 Likert-scale items and 6 open-ended items. Through reviewing the computed expert prior distributions, we identified 31 items that indicated considerable prior probability of “low endorsement” or showed largely inconsistent expert ratings as reflected by “wider” prior curves. Following this screening process, an in-depth discussion took place, during which written comments provided by experts were taken into consideration to reword items for improved clarity and revise the structure of the questionnaire to improve logical flow. The final version of the BLUE-Q is comprised of 3 parts (i.e., part 1 focuses on evaluations of the content and material of a blended learning program; part 2 focuses on the synchronous environment, and part 3 on the asynchronous environment) with a total of 23 Likert-scale items and 6 open-ended items. The final version of the BLUE-Q can be found in the Supplement 4.

## Discussion

### Key results

In this study, the content validity of the BLUE-Q was established through a Bayesian validation approach. Through this process, 31 items with poor probability were identified and removed, and the remaining items were slightly revised for phrasing and sequence to ensure clarity and logical flow.

### Interpretation

This article demonstrates that incorporating expert assessment for the purpose of Bayesian questionnaire validation is a straightforward process that can be conducted through relatively simple analyses. By observing the distinctive and informative prior distributions developed through analyzing the relative degree of agreement in expert ratings, we could identify which items could be immediately removed and which items could be kept after further revision. In so doing, the tool was refined tremendously. It can now be used to (1) evaluate the usability of blended learning programs deployed across the field of health professions education, (2) systematize program evaluations for blended learning programs, and (3) facilitate rigorous comparison between different blended learning programs.

### Comparison with previous studies

Traditional approaches to validating questionnaires suggest having a minimum sample of 100 to 250 participants [10]. Through the example of the BLUE-Q, we demonstrate the applicability of using a small sample size (i.e., 10 experts) for this Bayesian process. In so doing, the process for validating questionnaires becomes substantially less costly and time-consuming.

### Limitations

This study focuses on presenting the Bayesian questionnaire validation process for content validity. Content validity is only one type of validity measurement, albeit one of the most important types of validity in the design process of questionnaires. The Bayesian approach taken, though sound, also has certain limitations. Firstly, this approach relies on expert judgment, meaning prior data may be biased. It is essential, thus, to ensure that a wide range of experts are included in Bayesian studies and that diverse perspectives are accounted for when establishing prior data.

Additionally, if expert judgment is highly uncertain, prior distributions can sometimes skew results. This can be addressed by either recruiting more participants or recognizing that the high variability around an item may hinder its overall credibility or utility for a brief questionnaire. Finally, this study only considers faculty members’ perspectives, not learners. Future studies will validate the BLUE-Q with learners and collect further reliability and validity evidence.

### Generalizability

Though this study was conducted with faculty members in one Canadian-based institution, the BLUE-Q and the Bayesian validation approach may both prove useful and relevant for health professions education scholars and evaluators globally. The validation process can be implemented across institutions and countries to easily and quickly ensure that tools prove valid in terms of content for different contexts. The BLUE-Q, additionally, provides a comprehensive approach to evaluating programs and can be implemented across institutions to ensure rigor in quality improvement and program evaluation across health professions education.

### Suggestions

While more work is needed to further validate the BLUE-Q (e.g., establishing construct-validity, criterion-validity, and collecting reliability evidence through the application of the tool with actual health professional learners) [6], and this will be the focus of future studies, the Bayesian validation approach serves as a useful and efficient approach to gauge the perspectives of experts in order to refine tools. Furthermore, it is important to re-state that to ensure questionnaires are appropriate, well-designed, and clear to users, they must go through a rigorous validation process. However, this process can be complex, costly, and time-consuming. In turn, many scholars adopt previously validated questionnaires for their evaluation purposes, however, even in these cases, re-validation may often be necessary if a questionnaire is being deployed in a setting or with a population different from those that the tool was originally validated with. By demonstrating the simplicity of this Bayesian validation approach, we suggest that it should be used more widely across the field of health professions education to assist scholars in quickly and easily validating educational tools (e.g., for evaluating learners, educators, programs, or completing needs assessments).

### Conclusion

We believe that Bayesian questionnaire validation using expert priors is an important research method that can be easily integrated across studies in health professions education. In so doing, researchers can improve the content validity of newly developed instruments, modified instruments, and instruments that were previously validated but are now being deployed in novel settings or in different target populations than those that were originally used to validate the instrument. Our example with the BLUE-Q demonstrates how this can be done without conducting a large-scale, time-consuming, and resource-intensive validation study. In addition to the importance of this Bayesian approach, we highlight the potential of the newly validated BLUE-Q in bringing rigor to blended learning program evaluation across health professions education.

## Figures and Tables

**Figure f2-jeehp-21-31:**
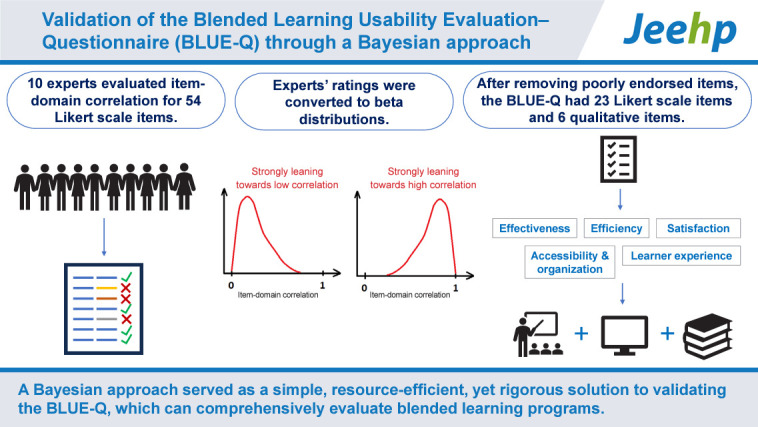


**Table 1.** Expert endorsements for the first 8 items of the BLUE-Q measuring “effectiveness” of course content and material

Item measuring ‘effectiveness’ of the course content and material	r_1_	r_2_	r_3_	r_4_	r_5_	r_6_	r_7_	r_8_	r_9_	r_10_	Alpha (sum)	Beta (# of raters minus Alpha)
1. The course content provided me with new knowledge or helped me clarify or deepen my understanding of the content at hand.	1	1	1	0.75	0.75	0.75	1	0.75	0.75	1	8.75	1.25
2. The course content helped me gain new skills or strengthen previously acquired skills.	0.5	1	1	0.5	0.75	0.75	1	0.25	0.75	1	7.5	2.5
3. The content taught in this course reflects the learning objectives or goals that were discussed in the first session of this course or in the course syllabus.	0.75	1	0.75	0.5	1	1	0.75	NA	1	0	6.75	2.25
4. The assessments (e.g., projects, tests) in this course were helpful for my learning.	0.75	0.5	0.75	0.25	0.75	0.75	1	0.25	1	0.75	6.75	3.25
5. The assessments in this course were fair.	0.25	0	0.25	0	0.75	0.75	0.5	0	1	0	3.5	6.5
6. Overall, I learned a lot from this course.	0.5	0.75	1	0.75	0.25	0.75	0.75	0.25	1	1	7	3
7. The instructor for this course assisted in my learning of the content.	0.75	0	0.75	0.25	NA	0.75	0.5	1	0.75	0.25	5	4
8. This course provided opportunities to learn via different styles (e.g., visual, aural, social).	0.75	0	0.75	0.75	0.25	0.5	0.25	0	1	0.75	5	5

The columns Alpha and Beta indicate statistical indices required to compute the shape parameters of the beta prior distribution, where Alpha represents the sum of all numerical expert ratings per item and Beta is the number of experts that provided a rating. BLUE-Q, Blended Learning Usability Evaluation–Questionnaire; NA, not answered.

## Footnotes

**Data availability**
Data files are available from Harvard Dataverse: https://doi.org/10.7910/DVN/0EF5KI Dataset 1. Rater’s response data on the 54 items of this study.

## Supplementary materials

Supplementary files are available from Harvard Dataverse: https://doi.org/10.7910/DVN/0EF5KI

Supplement 1. Description of the Blended Learning Usability Evaluation–Questionnaire (BLUE-Q).

Supplement 2. Statistical explanation of the Bayesian validation approach.

Supplement 3. Beta prior distributions summarizing expert endorsements for all 54 original items of the Blended Learning Usability Evaluation–Questionnaire (BLUE-Q).

Supplement 4. Final version of the Blended Learning Usability Evaluation–Questionnaire (BLUE-Q).

Supplement 5. Audio recording of the abstract.
