# Supplementary material for: Validation of the Blended Learning Usability Evaluation–Questionnaire (BLUE-Q) through an innovative Bayesian questionnaire validation approach
Source: J Educ Eval Health Prof. 2024 Nov 7;21:31. doi: 10.3352/jeehp.2024.21.31 (PMC11894031; doi:10.3352/jeehp.2024.21.31)
Supplement: Supplementary file 2 — Supplement 1. Description of the Blended Learning Usability Evaluation–Questionnaire (BLUE-Q). [file jeehp-21-31-suppl1.docx]

**Supplement 1.** Description of the Blended Learning Usability Evaluation–Questionnaire (BLUE-Q)

**What is the BLUE-Q**

The Blended Learning Usability Evaluation–Questionnaire (BLUE-Q) is an instrument that has been developed and designed by researchers at McGill University. The purpose of the BLUE-Q is to: (1) evaluate the usability of blended learning programs (BLPs), specifically those deployed across medical and health sciences education; (2) systematize program evaluations for BLPs; and (3) facilitate rigorous comparison between different BLPs.

The BLUE-Q is divided into 3 parts: the first assesses pedagogical usability (i.e., the program content, learning objectives, and experience of learners with their instructors); the second assesses the synchronous learning aspects of the program (i.e., face-to-face learning components that could be done in-person or via video-conferencing software); and the third assesses the asynchronous learning aspects of the program (i.e., the online learning tasks and learning management system). In total, the BLUE-Q has 29 items, 23 of which are quantitative (i.e., questions that collect responses on a 5-point Likert scale) and 6 of which are qualitative (i.e., questions that collect open-ended responses).

This instrument was developed through a rigorous 3-phase research design encompassing a large-scale scoping review, a qualitative descriptive study, and a Bayesian questionnaire validation approach. Through the first 2 phases of this approach, a comprehensive conceptual framework around usability evaluation for BLPs in medical and health sciences education was developed. This conceptual framework highlights that usability is a multidimensional construct with 6 main sub-domains: effectiveness, efficiency, satisfaction, accessibility, organization, and user experience. The specific definition for each sub-domain is provided Table 1. The BLUE-Q has items that span across all 6 sub-domains of usability, making it the first comprehensive instrument to evaluate usability in its totality across BLPs.

**Who should use the BLUE-Q**

The BLUE-Q should be given to learners taking part in a BLP, particularly BLPs given across the field of medical and health sciences education. This includes programs given to undergraduate, graduate, continuing professional development, faculty development, and any other program that adopts a blended learning approach to education. Note that we define BLPs as any program that offers education using a combination of synchronous (i.e., in-person or videoconferencing) and asynchronous (e.g., use of online modules). We consider programs to be truly blended when asynchronous approaches are limited to 30%–79% of the program duration.

**When should the BLUE-Q be used in program evaluations?**

We recommend that this instrument be given to learners at 2 time-pints: at the 33% completion time-point of the program and at the end of a program. The first application of this instrument will be useful in gauging early insight into the learner experience with the program. It will enable instructors and program designers to understand if and where changes in the program could or should be made. As the tool is applied early into the program, it may be possible to adapt the BLP slightly to better assist learners in the educational process if the need is apparent. This second application of the instrument will allow for an understanding of how learner perceptions of BLP usability have changed over the program. For example, if a BLP runs for 12 weeks, learners should be asked to complete the BLUE-Q at the 4-week mark and 12^th^ week of the program.

**How do you score the BLUE-Q**

The quantitative items are all scored on a 5-point Likert scale where strongly disagree equals 1 and strongly agree equals 5. Likert-scale items should be summed for each part for each student. The average of the sum scores should then be calculated. When the average score is 65% or less for any part of the questionnaire (i.e., learners give an average score of 32.5 for part 1 of the BLUE-Q; 19.5 for part 2; and 22.75 for part 3), then a moderate to serious problem with the BLP might exist and requires attention. When this threshold is reached for any parts of the BLUE-Q, educators and program evaluators should review items with the lowest scores and conduct a content analysis on the 2 qualitative items for those parts.

**Table 1.** Usability sub-domain definitions

| **Domain** | **Definition** |
| --- | --- |
| **Effectiveness** | Accuracy, completeness, and ease with which learners achieve learning objectives (e.g., gaining knowledge and/or skills). |
| **Efficiency** | Resources such as time, human effort, money, materials, used by learners, and their overall program engagement to achieve learning objectives. |
| **Satisfaction** | Positive or negative emotions and learners’ motivation to achieving learning objectives. |
| **Accessibility & organization** | Ease-of-access to and convenience of learning modalities and materials across time/space (taking into account technological literacy, limitations, and need for accommodating special needs of learners). Content and learning management systems designed and stylized in a clear, logical, easy-to-navigate manner. |
| **User experience** | Change in the perspectives of learners, faculties, and staff, including consideration of emotions and feelings beyond satisfaction. |
| **Pedagogical usability** | Appropriateness of course content and materials, with focus on reliability, relevance, understandability, engagingness, and adaptability of the content for learners. |
