# Supplementary material for: Validation of the Blended Learning Usability Evaluation–Questionnaire (BLUE-Q) through an innovative Bayesian questionnaire validation approach
Source: J Educ Eval Health Prof. 2024 Nov 7;21:31. doi: 10.3352/jeehp.2024.21.31 (PMC11894031; doi:10.3352/jeehp.2024.21.31)
Supplement: Supplementary file 3 — Supplement 2. Statistical explanation of the Bayesian validation approach. [file jeehp-21-31-suppl2.docx]

**Supplement 2.** Statistical explanation of the Bayesian validation approach

In statistics, Bayesian approaches aim at formalizing the process of knowledge updating by complementing existing knowledge (i.e., “prior information”) with newly emerging information [1]. Prior information can be leveraged from various sources including preliminary data, evidence synthesis from the literature, as well as expert knowledge. To leverage prior information through aggregating expert assessments for questionnaire validation, a Bayesian inference process is necessary [2]. This process entails prior information being obtained and statistically processed. When prior information is retrieved from individuals (i.e., knowledge experts), this step is typically referred to as “prior elicitation.” To formally summarize the overall information obtained, established probability distributions are used to encode the level of uncertainty of the elicited information. The resulting probability function is called a “prior distribution.” Step 2 requires the collection of new information, for example, data generated by a research study investigating the same phenomenon or question that has been captured with the prior distribution. This emerging evidence is statistically expressed as another probability distribution which is referred to as the likelihood function. The final step entails the combination of the obtained prior information with the new information. This step can also be described as information updating. Essentially, this updating is achieved by multiplying the prior distribution with the likelihood function, yielding a new statistical distribution that is referred to as the posterior distribution.

In the context of questionnaire development and validation, the first step alone in the Bayesian inference process offers an effective formal approach to leverage expert knowledge for assessing content validity of questionnaires (i.e., if items in a questionnaire are understandable by end-users, if the items appear meaningful and comprehensive for the intended measurement purposes, and if there is sufficient item-domain correlation). In essence, through the Bayesian questionnaire validation approach, experts are asked to provide a rating that expresses their level of endorsement (i.e., if they perceive that the respective item is appropriate for measuring the specific “latent trait” or in other words: “construct,” “factor,” or “domain” it was designed to measure), for each item of the questionnaire. Consistently low ratings indicate that an item needs to be revised or entirely removed from the tool.

**References**

1. Donovan TM, Mickey RM. Bayesian statistics for beginners: a step-by-step approach. Oxford University Press; 2019.

2. Rizzo DB, Blackburn MR. Harnessing expert knowledge: defining Bayesian network model priors from expert knowledge only—prior elicitation for the vibration qualification problem. IEEE Syst J 2019;13:1895-1905. <https://doi.org/10.1109/JSYST.2019.2892942>
