# Supplementary material for: Validation of the Blended Learning Usability Evaluation–Questionnaire (BLUE-Q) through an innovative Bayesian questionnaire validation approach
Source: J Educ Eval Health Prof. 2024 Nov 7;21:31. doi: 10.3352/jeehp.2024.21.31 (PMC11894031; doi:10.3352/jeehp.2024.21.31)
Supplement: Supplementary file 4 — Supplement 3. Beta prior distributions summarizing expert endorsements for all 54 original items of the Blended Learning Usability Evaluation–Questionnaire (BLUE-Q). [file jeehp-21-31-suppl3.docx]

**Supplement 3.** Beta prior distributions summarizing expert endorsements for all 54 original items of the Blended Learning Usability Evaluation–Questionnaire (BLUE-Q)


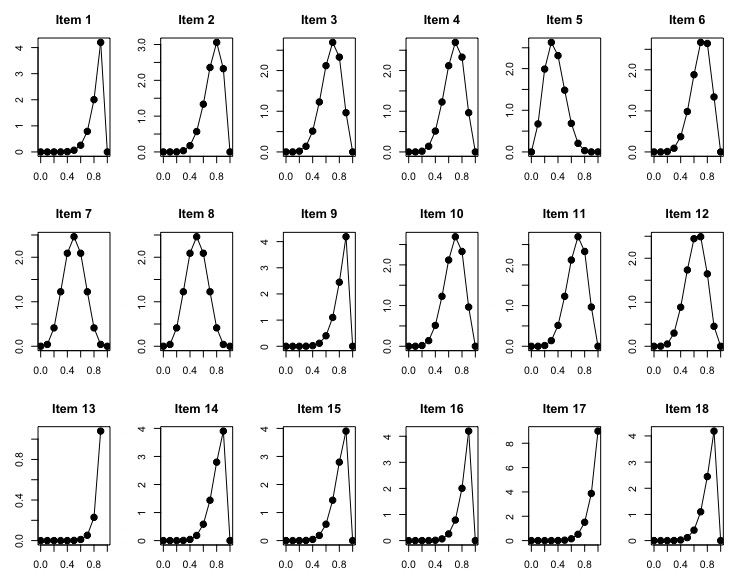


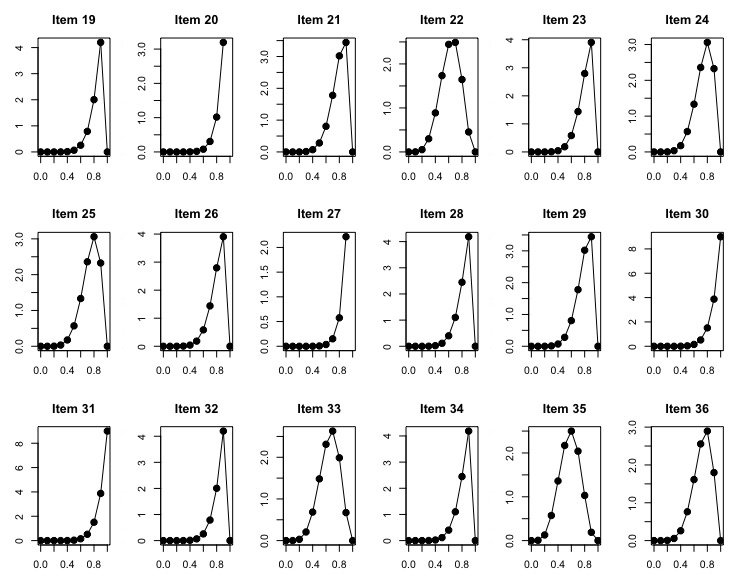

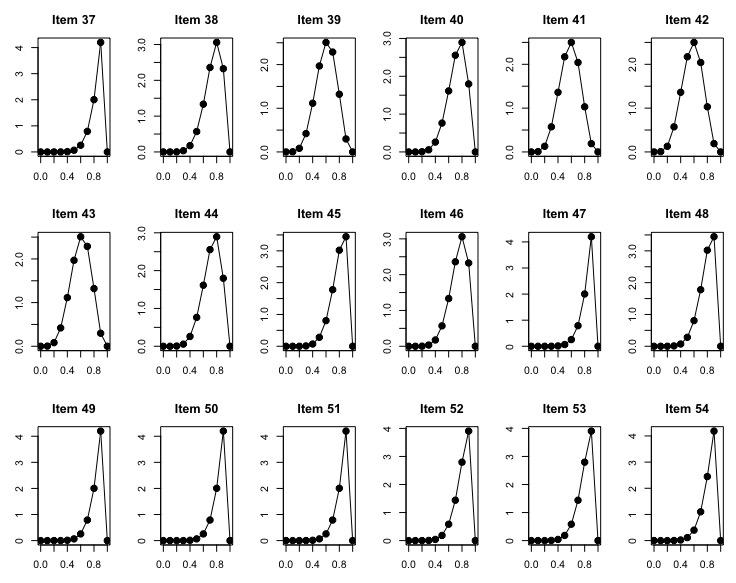


Original items 1–18 pertain to part 1 of the questionnaire (i.e., usability of the content and materials); items 19–32 pertain to part 2 of the questionnaire (i.e., the usability of the synchronous learning component of the program); and items 33–54 pertain to part 3 of the questionnaire (i.e., the usability of the asynchronous learning component of the program). In terms of further breakdown by domain: items 1–8, 19–22, and 33–39 are associated with effectiveness; items 9–12, 23–25, and 40–44 are associated with efficiency; items 13–15, 26–28, and 45–48 are associated with satisfaction; and items 16–18, 29–32, and 49–54 are associated with accessibility and organization.
