# Supplementary material for: Validation of the Blended Learning Usability Evaluation–Questionnaire (BLUE-Q) through an innovative Bayesian questionnaire validation approach
Source: J Educ Eval Health Prof. 2024 Nov 7;21:31. doi: 10.3352/jeehp.2024.21.31 (PMC11894031; doi:10.3352/jeehp.2024.21.31)
Supplement: Supplementary file 5 — Supplement 4. Final version of the Blended Learning Usability Evaluation–Questionnaire (BLUE-Q). [file jeehp-21-31-suppl4.docx]

**Supplement 4.** Final version of the Blended Learning Usability Evaluation–Questionnaire (BLUE-Q)

**Part 1. Pedagogical usability (content/materials)**

**Starting prompt:** The following questions are focused on your thoughts and experiences in relation to the content and materials (e.g., theories, ideas, perspectives, tasks, and assignments) that you engaged with in this program.

| **Items** | **Strongly disagree** | **Disagree** | **Neutral** | **Agree** | **Strongly agree** |
| --- | --- | --- | --- | --- | --- |
| **Effectiveness** |  |  |  |  |  |
| 1. The content taught in this course helped me gain new knowledge (i.e., facts or information) and/or strengthen previously acquired knowledge. |  |  |  |  |  |
| 2. The content taught in this course helped me gain new skills (i.e., ability to perform specific tasks) and/or strengthen previously acquired skills. |  |  |  |  |  |
| 3. The content taught in this course corresponds to the learning objectives discussed in the introduction of the course and/or course syllabus. |  |  |  |  |  |
| 4. The assessments (e.g., projects, assignments, and tests) in this course were helpful for my learning. |  |  |  |  |  |
| 5. Overall, I learned a lot from this course. |  |  |  |  |  |
| **Efficiency** |  |  |  |  |  |
| 6. The amount of work required for this course was manageable. |  |  |  |  |  |
| 7. The instructor was available to answer my questions. |  |  |  |  |  |
| **Satisfaction** |  |  |  |  |  |
| 8. I enjoyed learning the content in this course. |  |  |  |  |  |
| 9. I was motivated to learn the content in this course. |  |  |  |  |  |
| **Accessibility** & **organization** |  | |  |  |  |
| 10. The content of this course was delivered in a way that made sense to me. |  |  |  |  |  |

The following questions are asked in a short-answer format. Responses should be examined if the averaged sum of the quantitative responses falls below 65%.

**User experience**

1. Please share your overall thoughts in relation to the content or materials in your program.

**Future improvement**

2. Do you have any suggestions to improve the content or materials for future iterations of the program?

**Part 2. Synchronous learning environment**

**Starting prompt:** The following questions apply to the synchronous “face-to-face” component of your blended learning program. This is the component of the program that happens in real time, where the learners and instructors either met online through a video-conferencing software or in person.

| **Items** | **Strongly disagree** | **Disagree** | **Neutral** | **Agree** | **Strongly agree** |
| --- | --- | --- | --- | --- | --- |
| **Effectiveness** |  |  |  |  |  |
| 1. Being face-to-face with the teacher helped me learn the course content. |  |  |  |  |  |
| **Efficiency** |  |  |  |  |  |
| 2. The amount of time we spent in the face-to-face component was appropriate. |  |  |  |  |  |
| **Satisfaction** |  |  |  |  |  |
| 3. I enjoyed the face-to-face component. |  |  |  |  |  |
| 4. I felt motivated to attend the face-to-face component. |  |  |  |  |  |
| **Accessibility** & **organization** |  |  |  |  |  |
| 5. The face-to-face component of this course was easy-to-access (e.g., the classroom was easy to find, or the online videoconferencing software was easy to download or access on my technological device). |  |  |  |  |  |
| 6. The material taught in the face-to-face component was well organized. |  |  |  |  |  |

The following questions are asked in a short-answer format. Responses should be examined if the averaged sum of the quantitative responses falls below 65%.

**User experience**

1. Please share your overall thoughts in relation to the synchronous learning aspects of your program.

**Future improvement**

2. Do you have any suggestions to improve the synchronous learning aspects for future iterations of the program?

**Part 3. Asynchronous learning environment**

**Starting prompt:** The following questions apply to the asynchronous online learning component of your blended learning program. This is the part of your program that is completely online and at your own time (e.g., watching online modules or accessing learning management systems such as Moodle, Blackboard, Avenue to Learn, and MyCourses).

| **Items** | **Strongly disagree** | **Disagree** | **Neutral** | **Agree** | **Strongly agree** |
| --- | --- | --- | --- | --- | --- |
| **Effectiveness** |  |  |  |  |  |
| 1. The asynchronous online learning tasks (e.g., online modules) helped me learn. |  |  |  |  |  |
| **Efficiency** |  |  |  |  |  |
| 2. The amount of time I spent completing asynchronous online learning tasks was appropriate. |  |  |  |  |  |
| **Satisfaction** |  |  |  |  |  |
| 3. I enjoyed the asynchronous online learning tasks. |  |  |  |  |  |
| 4. I felt motivated to complete the asynchronous online learning tasks. |  |  |  |  |  |
| **Accessibility** & **organization** |  |  |  |  |  |
| 5. The learning management system (e.g., Moodle/Blackboard) for this course was easy-to-access on my technological devices. |  |  |  |  |  |
| 6. The learning management system was easy to navigate (i.e., I was able to quickly figure out how to access the course material on the online learning management system). |  |  |  |  |  |
| 7. The learning management system was well organized. |  |  |  |  |  |

The following questions are asked in a short-answer format. Responses should be examined if the averaged sum of the quantitative responses falls below 65%.

**User experience**

1. Please share your overall thoughts in relation to the asynchronous online learning aspects of your program.

**Future improvement**

2. Do you have any suggestions to improve the asynchronous online learning aspects for future iterations of the program?
